# Supplementary material for: The Arabidopsis MIK2 receptor elicits immunity by sensing a conserved signature from phytocytokines and microbes
Source: Nat Commun. 2021 Sep 17;12:5494. doi: 10.1038/s41467-021-25580-w (PMC8448819; doi:10.1038/s41467-021-25580-w)
Supplement: Supplementary file 1 — Supplementary Information [file 41467_2021_25580_MOESM1_ESM.pdf]

Supplementary information

Immune elicitation by sensing the conserved signature from phytocytokines and microbes via *Arabidopsis* MIK2 receptor

Shuguo Hou, Derui Liu, Shijia Huang, Dexian Luo, Zunyong Liu, Qingyuan Xiang, Ping Wang, Ruimin Mu, Zhifu Han, Sixue Chen, Jijie Chai, Libo Shan, and Ping He

Contents

Supplementary Figure 1. Activation of the cytosolic kinase domain of MIK2 triggers specific responses.
Supplementary Figure 2. PIP1 treatments induce the expression of secreted peptide genes.
Supplementary Figure 3. SCOOPs are secreted, processed, and active in shoots and roots.
Supplementary Figure 4. SCOOP-triggered responses depend on MIK2 and overlap with those by activation of the MIK2 kinase domain.
Supplementary Figure 5. TAMRA-SCOOP12 triggers root growth inhibition in a MIK2-dependent manner.
Supplementary Figure 6. The SCOOP-triggered response depends on BAK1 and SERK4.
Supplementary Figure 7. *Fusarium* spp. strains encode active *SCOOP-LIKE (SCOOPL)* motifs.
Supplementary Figure 8. *Fo*5176 Δ*SCOOPL* mutants show enhanced virulence in *Arabidopsis*.
Supplementary Figure 9. *Comamonadaceae* bacteria encode active SCOOPLs.

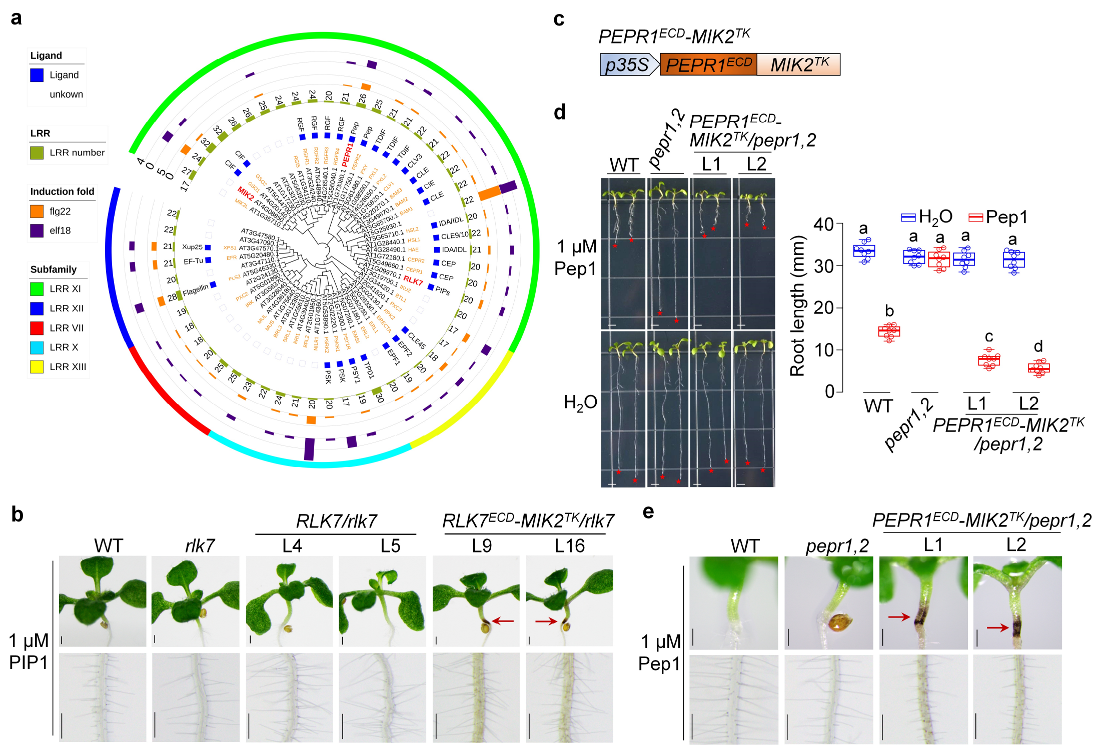

Supplementary Figure 1. Activation of the cytosolic kinase domain of MIK2 triggers specific responses.

a. *MIK2*, *RLK7*, and *PEPR1* are upregulated upon flg22 and elf18 treatments and phylogenetically related. Phylogenetic analysis and MAMP-induced expression of 56 LRR-RKs from the subfamily X (light blue curved line), XI (green curved line), XII (blue curved line), XIII (yellow curved line), and VII (red curved line). The LRR-RK full-length sequences were retrieved from NCBI (https://www.ncbi.nlm.nih.gov/) for MEGAX phylogenetic analysis using the neighbor-joining method with 1000 bootstrap replicates. The phylogenetic tree was displayed by iTOL v5 online software (https://itol.embl.de/). Blue squares indicate the cognate known ligands. Green bars with numbers indicate the number of LRRs for the corresponding LRR-RKs. Orange and purple bars indicate the induction levels of the cognate LRR-RK genes upon flg22 or elf18 treatment, respectively, according to the data from GENEVESTIGATOR V3. MIK2, RLK7*,* and PEPR1, used in this study, were highlighted in red.

b. Activation of the cytosolic kinase domain of MIK2 induces the brown roots and darkened hypocotyl-root junctions. Transgenic seedlings of *RLK7^{ECD}-MIK2^{TK}/rlk7*, but not WT or *RLK7/rlk7*, show the brown roots (bottom panel) and darkened hypocotyl-root junctions (top panel) upon PIP1 treatment. Seedlings of WT (Col-0), *rlk7*, two lines (L4 and L5) of *RLK7/rlk7*, and two representative lines (L9 and L16) of *RLK7^{ECD}-MIK2^{TK}/rlk7* were grown on ½MS plates with or without 1 µM PIP1for ten days. Red arrows indicate the hypocotyl-root junctions. Scale bar, 1 mm.

c. Diagram of the *PEPR1^{ECD}-MIK2^{TK}* chimeric receptor. The extracellular domain of *PEPR1* (*PEPR1^{ECD}*) was fused with the transmembrane and cytoplasmic kinase domain of *MIK2* (*MIK2^{TK}*) to generate the *PEPR1^{ECD}-MIK2^{TK}* chimeric receptor gene. The

PEPR1^{ECD}-MIK2^{TK} transgene under the control of a *35S* promoter was transformed into the *pepr1,2* mutant.

d, Activation of the cytosolic kinase domain of MIK2 inhibits root growth. Two representative lines (L1 and L2) of *PEPR1^{ECD}-MIK2^{TK}/pepr1,2* transgenic seedlings showed more severe root growth inhibition to Pep1 treatment than WT plants. Seedlings were grown on ½MS plates with or without 1 µM Pep1 for ten days (left). Seedling root length is shown in box plots on the right. Red stars indicate the root tips. Box plots show the first and third quartiles as bounds of box, split by the medians (lines), with whiskers extending 1.5-fold interquartile range beyond the box, and minima and maxima as error bar. Scale bar, 4 mm. Different letters indicate a significant difference with others ($P < 0.05$, One-way ANOVA followed by Tukey's test, $n = 8$).

e. Transgenic seedlings of *PEPR1^{ECD}-MIK2^{TK}/pepr1,2*, but not WT, show the brown roots (bottom panel) and darkened hypocotyl-root junctions (top panel) upon Pep1 treatment. Seedlings of WT (Col-0), *pepr1,2*, and two lines (L1 and L2) of *PEPR1^{ECD}-MIK2^{TK}/pepr1,2* were grown on ½MS plates with or without 1 µM Pep1 for ten days. Red arrows indicate the hypocotyl-root junctions. Scale bar, 1 mm.

The experiments in **b**, **d**, and **e** were repeated three times with similar results.

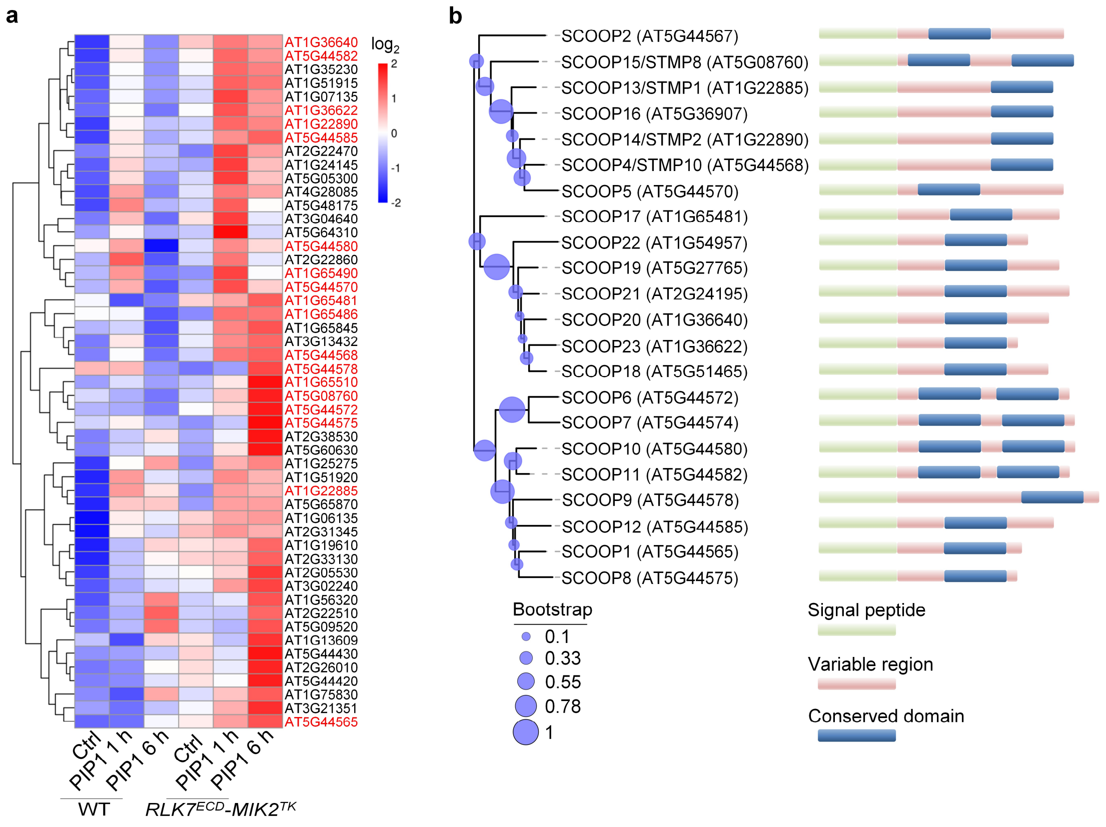

Supplementary Figure 2. PIP1 treatments induce the expression of secreted peptide genes.

a. Genes encoding SCOOPs, STMPs, and small secreted peptides are induced by PIP1 treatment in *RLK7^{ECD}-MIK2^{TK}/rlk7* transgenic plants. Ten-day-old seedlings grown on ½MS plates were treated with 1 μM PIP1 for 0, 1, and 6 h for RNA-Seq analyses. Heatmap represents transcript levels of secreted peptide genes. The original means of gene transcript levels represented by FPKM (Fragments Per Kilobase of exon model per Million mapped fragments) values were subjected to data adjustment by $\log_2$ transformation using the OmicStudio (https://www.omicstudio.cn/tool) for the heatmap. *SCOOP* and *STMP* genes are highlighted in red.

b. The phylogenetic analysis and domains of SCOOP/STMP proteins. Signal peptide, variable region, and conserved domain are indicated by colored rectangles. The phylogenetic tree was built by MEGAX using the neighbor-joining method with 1000 bootstrap replicates.

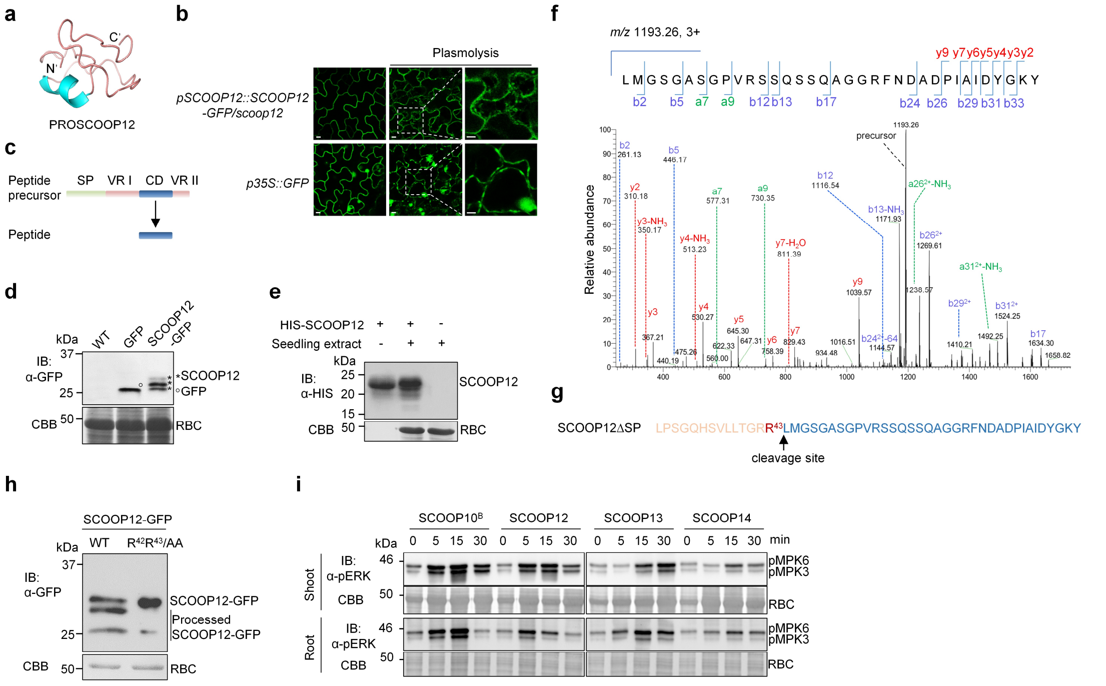

Supplementary Figure 3. SCOOPs are secreted, processed, and active in shoots and roots.

a. Three-dimensional structure of SCOOP12 (without signal peptide) predicted by I-TASSER (https://zhanglab.ccmb.med.umich.edu/I-TASSER/). Helix structure is shown in cyan, and the rest in orange.

b. SCOOP12 is localized to the plasma membrane and apoplasts. Detached leaves from two-week-old soil-grown *p35S::GFP/*WT and *pSCOOP12::SCOOP12-GFP/scoop12* transgenic plants were treated without (left panel) or with 5% NaCl (right two panels) for 3 min for plasmolysis, and observed under a confocal laser scanning microscope with 472 nm excitation. The right panel shows the zoom-in view from the dashed area in the middle panel. Scale bar, 10 μm.

c. Diagram of secreted peptide processing in plants. A peptide precursor containing an N-terminal signal peptide (SP), variable region (VR), and a C-terminal conserved domain (CD) is processed in plants to generate an active peptide.

d. Multiple bands were detected for SCOOP12-GFP in *Arabidopsis* transgenic plants. Total proteins isolated from seven-day-old WT, *pSCOOP12::SCOOP12-GFP/scoop12*, and *p35S::GFP* transgenic seedlings were subjected to immunoblotting with α-GFP antibodies (top panel), and the protein loading is shown by CBB staining for RBC (bottom panel).

e. HIS-SCOOP12 proteins are processed by protein extracts from *Arabidopsis* seedlings. HIS-SCOOP12 proteins (5 μg) were incubated with 10 μL of protein extraction buffer or protein extracts isolated from ten-day-old *Arabidopsis* WT seedlings for 5 h at room temperature. The reaction products were subjected to immunoblotting with α-HIS antibodies (top panel), and the protein loading from seedling extracts is shown by CBB staining for RBC (bottom panel).

f. MS/MS spectrum of a triple charged peptide of m/z 1193.26 corresponding to the C-

terminus of SCOOP12. MS/MS analysis of *in vitro* processed proteins in **e** was performed**.** The MS/MS spectrum was exported from the raw data file using Xcalibur Quabrower and annotated with the assistance of Mascot and MS Product.

g. The cleavage site of SCOOP12 identified by LC-MS/MS. The SCOOP12 sequence without signal peptide (SP) is marked with the cleavage site after R^{43}.

h. $R^{42}R^{43}$ residues are required for SCOOP12 proteolysis. Protoplasts from leaves of four-week-old WT plants were transfected with SCOOP12- or SCOOP12$^{R42R43/AA}$-GFP and incubated for 8 h before protein extraction. Protein extracts were subjected to IB with α-GFP antibodies (top panel), and CBB staining for RBC as loading controls (bottom panel).

i. SCOOP peptides induce MAPK activation in shoots and roots. Ten-day-old WT seedlings grown in ½MS liquid medium were treated with or without 1 μM peptides for the indicated time. Shoots and roots were collected separately for protein isolation and MAPK activation assays by immunoblotting with α-pERK1/2 antibodies (top panel), and the protein loading is shown by CBB staining for RBC (bottom panel).

The experiments in **b** and **d**-**i** were repeated twice with similar results.

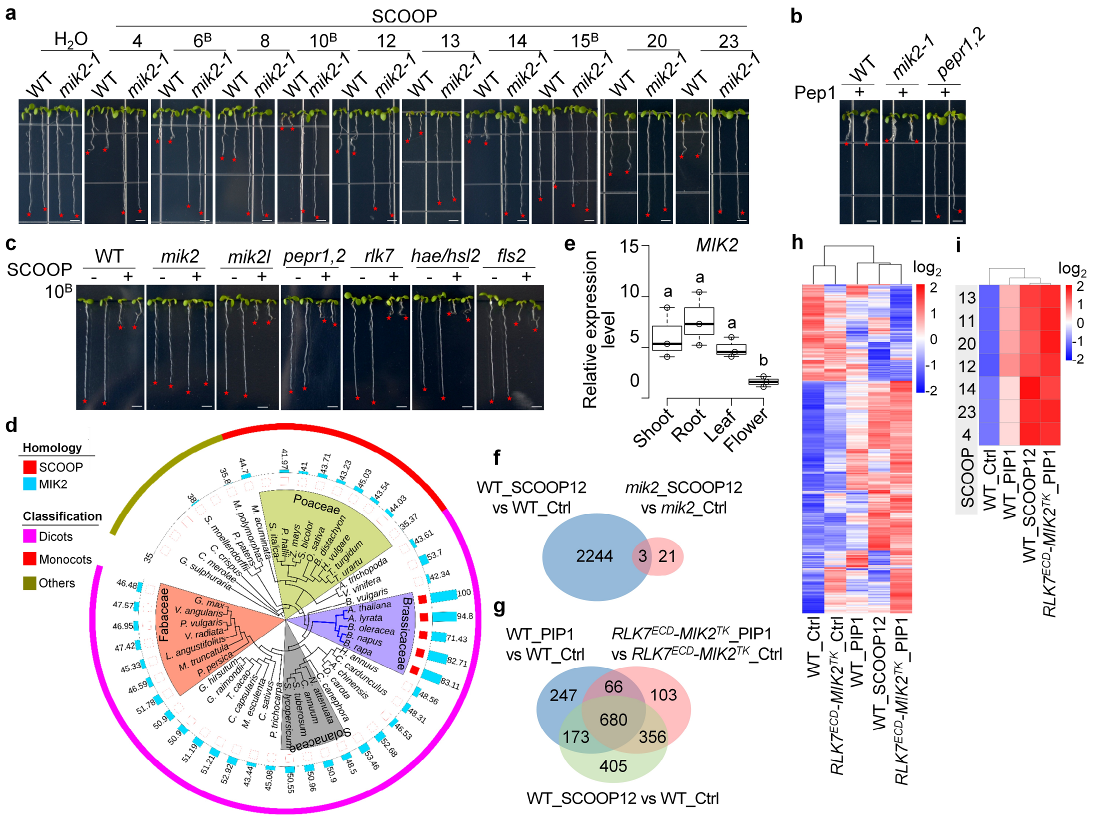

Supplementary Figure 4. SCOOP-triggered responses depend on MIK2 and overlap with those by activation of the MIK2 kinase domain.

a. SCOOP-triggered root growth inhibition is blocked in the *mik2-1* mutant. WT and *mik2-1* mutant seedlings were grown on ½MS plates with or without 1 µM SCOOP peptides for ten days.

b. The *mik2-1* mutant is sensitive to Pep1 as WT. WT, *mik2-1*, and *pepr1,2* seedlings were grown on ½MS plates with or without 1 µM Pep1 for ten days.

c. SCOOP10^{B} peptides trigger robust root growth inhibition in *mik2l*, *fls2*, *pepr1/2*, *rlk7*, and *hae/hsl2* mutants. Seedlings were grown on ½MS plates with or without 1 µM SCOOP10^{B} for ten days. Scale bar, 4 mm (**a-c**).

d. MIK2 orthologs in different plant species. MIK2 homologous protein sequences were blast-searched in NCBI using *Arabidopsis* MIK2 as a query, and the phylogenetic analysis was constructed based on the Rpb2 (RNA polymerase II second largest subunit) protein sequence from each species with MEGAX. Red, purple, and olive curved lines indicate monocots, dicots, and other plant classes, respectively; teal bars indicate the percentage of homology of MIK2 in different plant species (the percentages of amino acid identity to *Arabidopsis* MIK2 were labeled). Red squares indicate the presence of homologs of SCOOP. Peach, lime green, grey, and blue purple fans denote plant families *Fabaceae*, *Poaceae*, *Solanaceae*, and *Brassicaceae*, respectively.

e. Expression pattern of *MIK2* in different plant tissues. Total RNAs were isolated from the leaves of four-week-old soil-grown plants, shoots and roots of two-week-old plate-grown seedlings, and flowers of seven-week-old soil-grown plants for RT-qPCR analysis. The *MIK2* expression level was normalized to *UBQ10* and shown in bar charts.

Box plots show the first and third quartiles as bounds of box, split by the medians (lines), with whiskers extending 1.5-fold interquartile range beyond the box, and minima and maxima as error bar. Different letters indicate a significant difference ($P < 0.05$, One-way ANOVA followed by Tukey's test, $n = 3$).

f. SCOOP12-regulated gene transcription is largely abolished in *mik2-1*. Ten-day-old seedlings of the indicated genotypes (WT or *mik2-1*) were treated without or with 1 μM SCOOP12 for 1 h and mRNA was isolated for RNA-Seq analysis. Venn diagram shows the overlapping between two sets of differentially expressed genes.

g. SCOOP12-regulated genes largely overlap with MIK2 kinase domain-regulated genes in PIP1-treated $RLK7^{ECD}$-$MIK2^{TK}$/*rlk7* transgenic plants. Ten-day-old seedlings of the indicated genotypes (WT or $RLK7^{ECD}$-$MIK2^{TK}$/*rlk7*) were treated with H_2O or 1 μM PIP1 or SCOOP12 for 1 h for RNA-Seq analysis. Venn diagram shows the overlapping between different sets of differentially expressed genes.

h. SCOOP12-regulated genes are clustered together with MIK2 kinase domain-regulated genes in PIP1-treated $RLK7^{ECD}$-$MIK2^{TK}$/*rlk7* transgenic plants. Heatmap represents transcript levels of differentially expressed genes as indicated in **f**. The original means of gene transcript levels represented by FPKM (Fragments Per Kilobase of exon model per Million mapped fragments) values were subjected to data adjustment by log_2 transformation using the OmicStudio (https://www.omicstudio.cn/tool).

i. SCOOP12 upregulates *SCOOP* gene expression. The original means of *SCOOP* transcript levels represented by FPKM values were used to build a heatmap as indicated in **g**.

The experiments in **a-c,** and **e** were repeated three times with similar results.

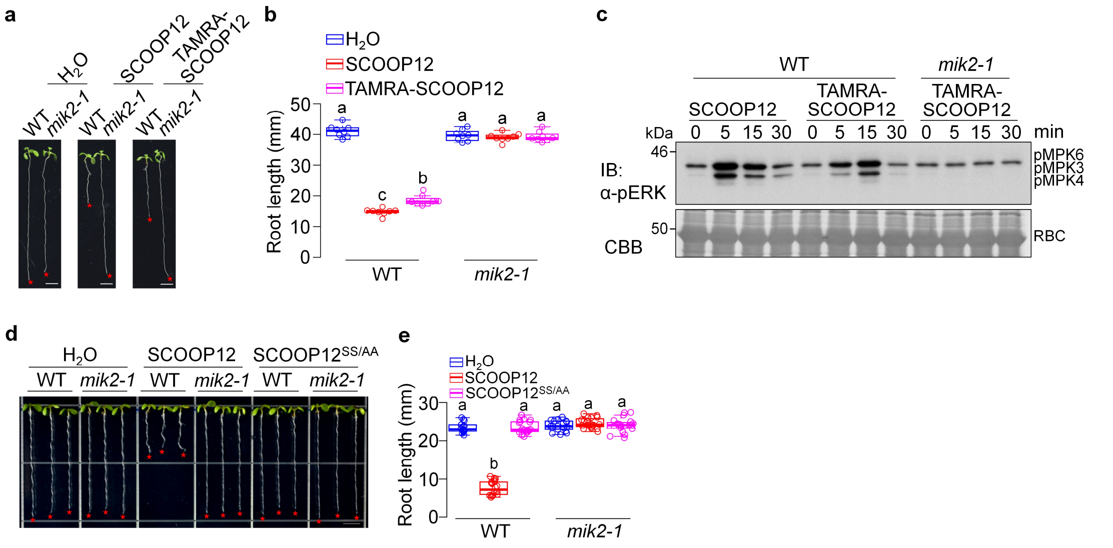

Supplementary Figure 5. TAMRA-SCOOP12 triggers root growth inhibition in a MIK2-dependent manner.

a, b. TAMRA-SCOOP12 peptides inhibit root growth in a MIK2-dependent manner. WT and *mik2-1* seedlings were grown on ½MS plates with or without 1 µM TAMRA-SCOOP12; scale bar, 4 mm (**a**). Quantification of root length of seedlings in **a** (**b**). Different letters indicate a significant difference with others (P < 0.05, One-way ANOVA followed by Tukey's test)

c. TAMRA-SCOOP12 peptides induce MAPK activation in a MIK2-dependent manner. Ten-day-old WT and *mik2-1* seedlings grown in ½MS liquid medium were treated with or without 1 µM SCOOP12 or TAMRA-SCOOP12 for the indicated time. The MAPK activation was analyzed by immunoblotting with α-pERK1/2 antibodies (top panel), and the protein loading is shown by CBB staining for RBC (bottom panel).

d, **e** SCOOP12$^{SS/AA}$ peptides are inactive to inhibit root growth. The assay and quantification of root length were performed as in **a**, **b** with or without 1 µM SCOOP12 or SCOOP12$^{SS/AA}$.

Box plots in **b** and **e** show the first and third quartiles as bounds of box, split by the medians (lines), with whiskers extending 1.5-fold interquartile range beyond the box, and minima and maxima as error bar. Different letters indicate a significant difference with others ($P < 0.05$, One-way ANOVA followed by Tukey's test, $n = 8$ in **b**; n = 18, 20, 20, 18, 20, 20 for plots from left to right in **e**).

The above experiments were repeated three times with similar results.

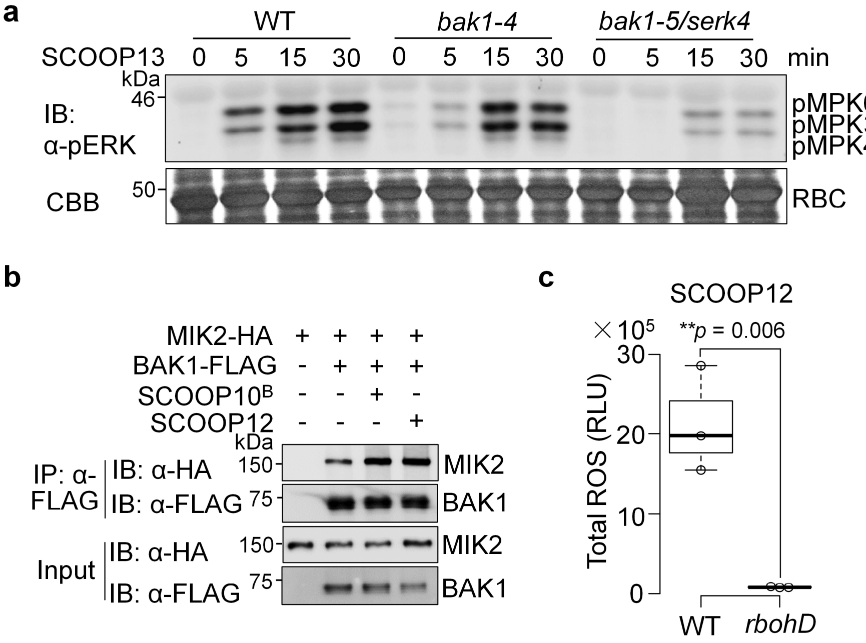

Supplementary Figure 6. The SCOOP-triggered response depends on BAK1 and SERK4.

a. SCOOP13-induced MAPK activation is compromised in *bak1-5/serk4.* Ten-day-old seedlings of WT, *bak1-4*, and *bak1-5/serk4* were treated with 100 nM SCOOP13 for the indicated time. The MAPK activation was analyzed by immunoblotting with a-pERK1/2 antibodies (top panel), and the protein loading is shown by CBB staining for RBC (bottom panel).

b. SCOOPs enhance the association of MIK2 and BAK1 in protoplasts. Protoplasts from WT plants were co-transfected with HA-tagged MIK2 (MIK2-HA) and FLAG-tagged BAK1 (BAK1-FLAG), or a control vector (Ctrl) and incubated for 10 h, followed by treatment with or without 1 μM $SCOOP^{B}$ or SCOOP12 for 15 min. Proteins were immunoprecipitated with α-FLAG agarose beads (IP: α-FLAG), followed by immunoblotting (IB) with α-HA or α-FLAG antibodies (top two panels). IB on the bottom two panels shows the input controls before IP.

c. SCOOP12-induced ROS production is blocked in the *rbohD* mutant. One-week-old seedlings grown on ½MS plates were treated with 100 nM SCOOP12, and ROS production as total RLUs were measured. Box plots show the first and third quartiles as bounds of box, split by the medians (lines), with whiskers extending 1.5-fold interquartile range beyond the box, and minima and maxima as error bar ($^{**}P < 0.01$, two-tailed Student's *t*-test, $n = 3$).

The above experiments were repeated three times with similar results.

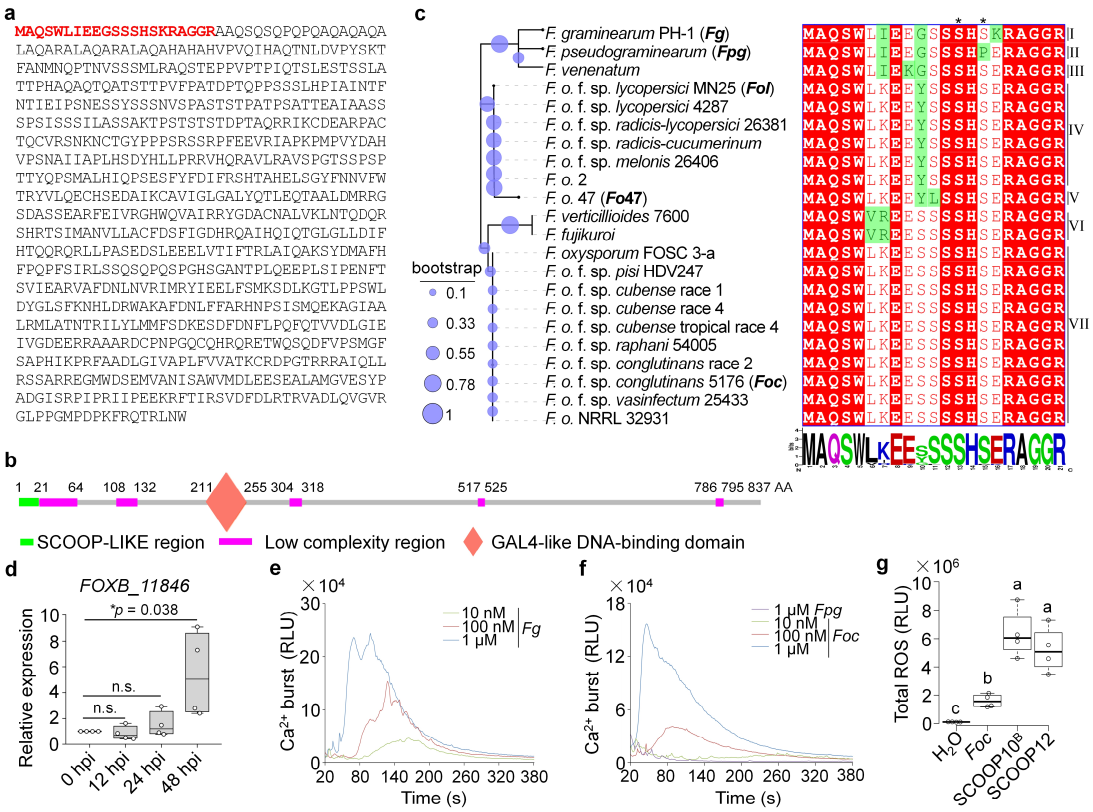

Supplementary Figure 7. *Fusarium* spp. strains encode active SCOOP-LIKE (SCOOPL) motifs.

a. A SCOOP-LIKE motif localizes in the N-terminus of FGSG_07177 in *F. graminearum*. The sequence from *F. graminearum* was downloaded from the NCBI website, and the SCOOPL sequence was marked in red.

b. Schematic diagrams of FGSG_07177 protein motifs. The predicated domains analyzed by the SMART database (https://smart.embl.de/) were highlighted with amino acid positions labeled on the top.

c. The SCOOPL motif is conserved in *Fusarium* species. The SCOOPL motifs were blast-searched in the NCBI and EnsemblFungi (http://fungi.ensembl.org/) database using *F. graminearum* SCOOPL as the reference, aligned using ClustalW and visualized in ESPript3 (espript.ibcp.fr). The SxS motif was marked with stars on the top. The consensus of SCOOPs was conducted with WebLogo (http://weblogo.berkeley.edu/logo.cgi). SCOOPL peptides from *Fg*, *Fpg*, *Foc*, *Fol*, and *Fo*47 (highlighted in bold) were synthesized for bioassays. The phylogenetic tree was constructed with MEGAX using neighbor-joining methods. The bootstrap values from 1000 replications are indicated on the branches.

d. *FOXB_11846* is induced during *Fo*5176 infection in *Arabidopsis*. *Fo*5176 spores ($1{\times}10^7$/mL) were inoculated onto three-week-old *Arabidopsis* seedlings. Fungal mRNAs were isolated at 0, 12, 24, and 48 h post-inoculation (hpi) and subjected to RT-qPCR analysis. The expression of *FOXB_11846* was normalized to that of *Fusarium GAPDH*, and the relative expression levels are shown in box plots (*$P < 0.05$, n.s., no significant differences, two-tailed Student's *t*-test, $n = 4$).

e, **f.** SCOOPL synthetic peptides from *Fg* and *Foc*, but not from *Fpg*, trigger the cytosolic Ca^{2+} increase. One-week-old transgenic seedlings expressing *p35S::Aquorin* grown on ½MS plates were pre-treated with coelenterazine in dark overnight, followed by treatment with or without peptides with the indicated concentrations for the continuous measurement of cytosolic Ca^{2+} concentration with a one-second interval for 10 min. The values for cytosolic Ca^{2+} concentration indicated as means of RLU are shown in box plots.

g. *Foc*SCOOPL induces a weaker ROS production than $SCOOP10^{B}$ and SCOOP12. One-week-old WT seedlings were treated with 1 µM *Foc*SCOOPL, 100 nM $SCOOP10^{B}$ or 100 nM SCOOP12, and ROS production was measured immediately with an interval of one second for 15 min. Total luminescence counts as RLU were calculated and shown in box plots for each treatment. Different letters indicate a significant difference with others ($P < 0.05$, One-way ANOVA followed by Tukey's test, $n = 4$).

Box plots in **d** and **g** show the first and third quartiles as bounds of box, split by the medians (lines), with whiskers extending 1.5-fold interquartile range beyond the box, and minima and maxima as error bar. The experiments in **d**-**g** were repeated three times with similar results.

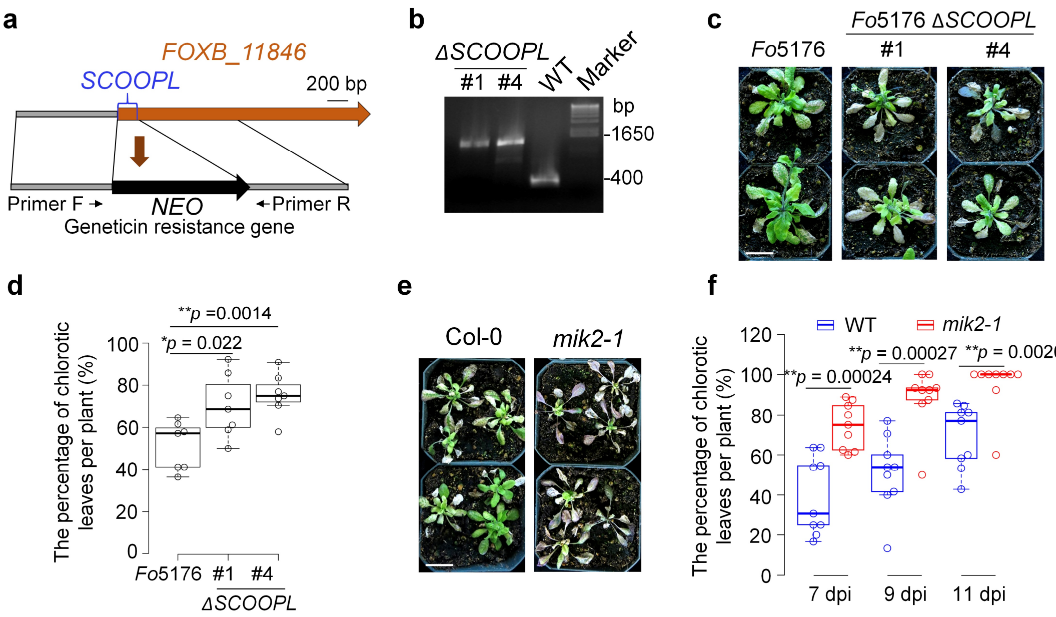

Supplementary Figure 8. *Fo*5176 Δ*SCOOPL* mutants show enhanced virulence in *Arabidopsis*.

a. Diagram of *SCOOPL* deletion from *FOXB_11846* in *Fo*5176. A gene replacement cassette containing a geneticin resistance gene (*NEO*) and *SCOOPL* flanking sequences was introduced into *Fo*5176 protoplasts for homologous recombination-based replacement of *SCOOPL* by *NEO*. Δ*SCOOPL* mutants were screened with 100 µg/L geneticin and identified by PCR amplification with primers labeled.

b. Identification of *Fo*5176 Δ*SCOOPL* mutants. PCR amplification was conducted by using genomic DNA of WT *Fo*5176 or Δ*SCOOPL* deletion mutants as templates and primers shown in **a**.

c. *Fo*5176 Δ*SCOOPL* mutants are more virulent than WT *Fo*5176 in *Arabidopsis*. Three-week-old *Arabidopsis* plants were inoculated with WT *Fo*5176 or Δ*SCOOPL* mutant strains at 1×10^7 spores/mL via the root-dipping method. The images were taken at 11 days post infection (dpi). Scale bar, 2 cm.

d. The disease scales of *Arabidopsis* plants infected with *Fo*5176 or *Fo*5176 Δ*SCOOPL*. The disease quantification was calculated with the ratio of chlorotic leaves in each plant (%) at 11 dpi.

e. The *mik2-1* mutant is markedly more susceptible to *Fo*5176 than WT plants. Three-week-old *Arabidopsis* plants were inoculated with *Fo*5176 at 1×10^7 spores/mL via root-dipping. The images were taken 11 dpi. Scale bar, 2 cm.

f. The disease scales of *Arabidopsis* plants infected with *Fo*5176. The disease quantification was calculated with the ratio of chlorotic leaves in each plant (%) at 7, 9, and 11 dpi.

Box plots in **d** and **f** show the first and third quartiles as bounds of box, split by the medians (lines), with whiskers extending 1.5-fold interquartile range beyond the box, and minima and maxima as error bar ($*P < 0.05$, $**P < 0.01$, one-way ANOVA followed by Tukey's test, $n = 7$ in **d**, $n = 9$ in **f**). The experiments in **b-f** were repeated twice with similar results.

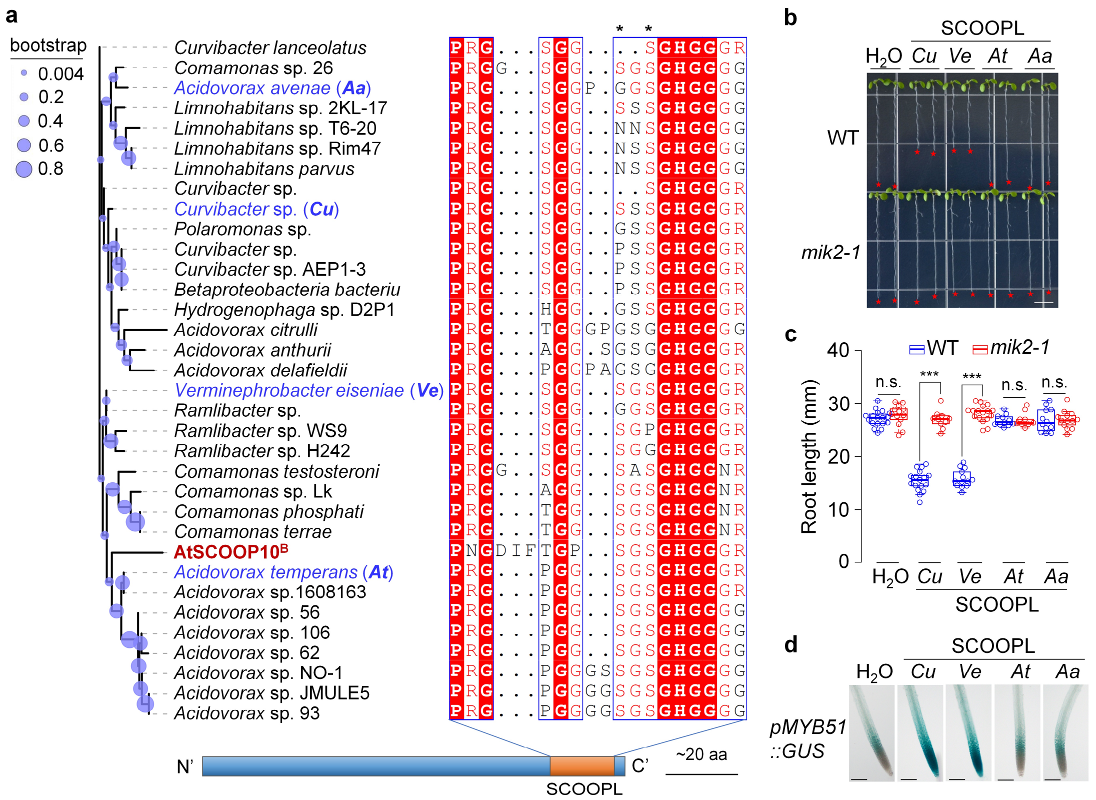

Supplementary Figure 9. ***Comamonadaceae*** **bacteria encode active SCOOPLs.**

a. Alignment and phylogenetic analysis of SCOOPL sequences in bacterial *Comamonadaceae*. SCOOPL sequences from different bacterial species in *Comamonadaceae* family were retrieved by blast-searching the NCBI database using *Arabidopsis* SCOOP10^{B} as the reference, aligned using ClustalW, and visualized in ESPript3 (espript.ibcp.fr). Conserved serine residues in the SxS motif are marked with stars on the top. SCOOPL peptides corresponding to *Cu*, *Ve*, *At*, and *Aa* (highlighted in blue) were synthesized for bioassays. The phylogenetic tree was constructed with MEGAX using neighbor-joining methods. The bootstrap values from 1000 replications are indicated on the branches. A diagram on the bottom showed the position of SCOOPL motif in an unknown functional protein in *Comamonadaceae* family bacteria.

b, **c.** Bacterial SCOOPL synthetic peptides inhibit root growth in a MIK2-dependent manner. WT and *mik2-1* mutant seedlings grown on ½MS plates with or without 1 μM peptides for ten days. Red stars indicate the root tips. Scale bar, 4 mm (**b**). Quantification of seedling root length in **b** (**c**). Box plots show the first and third quartiles as bounds of box, split by the medians (lines), with whiskers extending 1.5-fold interquartile range beyond the box, and minima and maxima as error bar. (****P* < 0.001, n.s., no significant differences, two-sided Student's *t*-test, *n* = 18, 13, 17, 10, 13, 17, 11, 9, 12, 13 for plots from left to right) (**c**).

d. Bacterial SCOOPLs induce the expression of *pMYB51::GUS* in roots. One-week-old transgenic seedlings carrying*pMYB51::GUS* grown on ½MS plates were treated with or without 1 μM bacterial SCOOPL peptides for 3 h and subjected to GUS staining and distaining followed by photographing under a stereomicroscope. Scale bar, 1mm.

The experiment was repeated three times with similar results.

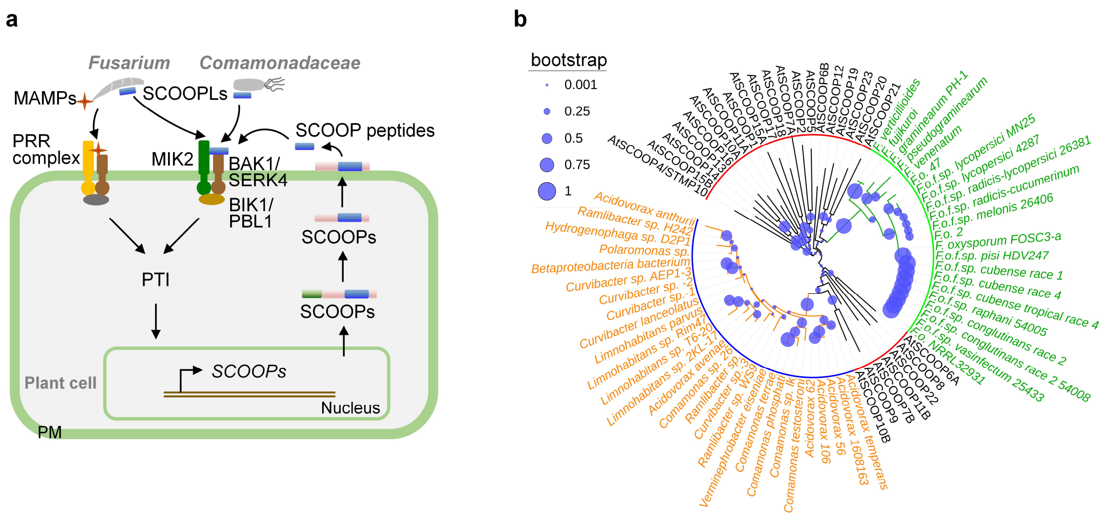

Supplementary Figure 10. A proposed model for SCOOP/SCOOPL-MIK2 functions and phylogenetic analysis of SCOOPs/SCOOPLs.

a. A proposed model for SCOOP/SCOOPL-MIK2 function in plant immunity. Upon pathogen exposure, plant PRRs sense MAMPs in activating plant pattern-triggered immunity (PTI). MIK2 as a PRR recognizes the MAMP SCOOPLs derived from fungal *F. oxysporum* and bacterial *Comamonadaceae* in activating PTI. PTI activation induces the production of plant SCOOPs, which are in turn perceived by MIK2 to amplify PTI, thereby collectively contributing plant resistance against fungal *F. oxysporum*. SCOOPs/SCOOPLs induce MIK2 association with BAK1/SERK4 coreceptors and relay signaling through BIK1/PBL1 RLCKs.

b. Phylogenetic analysis of SCOOP and SCOOPL peptides. The phylogenetic tree was constructed with MEGAX using neighbor-joining methods. The bootstrap values from 1000 replications are indicated on the branches.
