# Supplementary material for: The Arabidopsis MIK2 receptor elicits immunity by sensing a conserved signature from phytocytokines and microbes
Source: Nat Commun. 2021 Sep 17;12:5494. doi: 10.1038/s41467-021-25580-w (PMC8448819; doi:10.1038/s41467-021-25580-w)
Supplement: Supplementary file 3 — Description of Additional Supplementary Files [file 41467_2021_25580_MOESM3_ESM.pdf]

Description of Additional Supplementary Files

Supplementary Data 1: Description

Secreted peptide genes upregulated by PIP1 in RLK7-MIK2 transgenic plants.

Supplementary Data 2: Description

SCOOP12-regulated genes in WT and mik2-1, and PIP1-regulated genes in WT and RLK7-MIK2 transgenic plants.

Supplementary Data 3: Description

Primers used in this study.

Supplementary Data 4: Description

Peptides used in this study.
