# Supplementary material for: The Arabidopsis MIK2 receptor elicits immunity by sensing a conserved signature from phytocytokines and microbes
Source: Nat Commun. 2021 Sep 17;12:5494. doi: 10.1038/s41467-021-25580-w (PMC8448819; doi:10.1038/s41467-021-25580-w)
Supplement: Supplementary file 8 — Reporting Summary [file 41467_2021_25580_MOESM8_ESM.pdf]

nature research

Corresponding author(s): Ping He

Last updated by author(s): 08/13/2021

Reporting Summary

Nature Research wishes to improve the reproducibility of the work that we publish. This form provides structure for consistency and transparency in reporting. For further information on Nature Research policies, see Authors & Referees and the Editorial Policy Checklist.

Statistics

For all statistical analyses, confirm that the following items are present in the figure legend, table legend, main text, or Methods section.

n/a	Confirmed	
☐	☒	The exact sample size (*n*) for each experimental group/condition, given as a discrete number and unit of measurement
☐	☒	A statement on whether measurements were taken from distinct samples or whether the same sample was measured repeatedly
☐	☒	The statistical test(s) used AND whether they are one- or two-sided<br>*Only common tests should be described solely by name; describe more complex techniques in the Methods section.*
☐	☒	A description of all covariates tested
☐	☒	A description of any assumptions or corrections, such as tests of normality and adjustment for multiple comparisons
☐	☒	A full description of the statistical parameters including central tendency (e.g. means) or other basic estimates (e.g. regression coefficient) AND variation (e.g. standard deviation) or associated estimates of uncertainty (e.g. confidence intervals)
☐	☒	For null hypothesis testing, the test statistic (e.g. *F*, *t*, *r*) with confidence intervals, effect sizes, degrees of freedom and *P* value noted<br>*Give P values as exact values whenever suitable.*
☒	☐	For Bayesian analysis, information on the choice of priors and Markov chain Monte Carlo settings
☒	☐	For hierarchical and complex designs, identification of the appropriate level for tests and full reporting of outcomes
☒	☐	Estimates of effect sizes (e.g. Cohen's *d*, Pearson's *r*), indicating how they were calculated

Our web collection on statistics for biologists contains articles on many of the points above.

Software and code

Policy information about availability of computer code

Data collection	-Image collection for Leica TCS SP8 confocal laser scanning microscope: Leica Application Suite X (LAS X).<br>-Data collection for the root length: Image J (http://rsb.info.nih.gov/ij/).<br>-Data collection for ethylene concentration: Chromeleon 7.<br>-Visualization of protein structures: Pymol (The PyMOL Molecular Graphics System, Version 1.2r3pre, Schrödinger, LLC.) and I-TASSER (https://zhanglab.ccmb.med.umich.edu/I-TASSER/).
Data analysis	-Image analysis for Leica TCS SP8 confocal images: LAS X.<br>-Data analysis for MS/MS: Mascot Daemon (Matrix Science, London, UK; version 2.4.0).<br>-Data analysis for the binding kinetics in SPR assays: BIAevaluation (Version 4.1).<br>-Data analysis for ITC: MicroCal Origin 7.0.<br>-Data analysis for consensus of peptide sequences: WebLogo (http://weblogo.berkeley.edu/logo.cgi).<br>-Phylogenetic analysis: MEGAX.<br>-Statistical analysis: p values were calculated in Excel 2016 with a one-way ANOVA (ANalysis Of VAriance) with post-hoc Tukey HSD (Honest Significant Difference) test or two-tailed unpaired Student's t-test.

For manuscripts utilizing custom algorithms or software that are central to the research but not yet described in published literature, software must be made available to editors/reviewers. We strongly encourage code deposition in a community repository (e.g. GitHub). See the Nature Research guidelines for submitting code & software for further information.

Data

Policy information about availability of data

All manuscripts must include a data availability statement. This statement should provide the following information, where applicable:

- Accession codes, unique identifiers, or web links for publicly available datasets
- A list of figures that have associated raw data
- A description of any restrictions on data availability

Original data that support the findings of this study are available from the corresponding author upon request. Source data are provided with this paper.

Field-specific reporting

Please select the one below that is the best fit for your research. If you are not sure, read the appropriate sections before making your selection.

☒ Life sciences ☐ Behavioural & social sciences ☐ Ecological, evolutionary & environmental sciences

For a reference copy of the document with all sections, see nature.com/documents/nr-reporting-summary-flat.pdf

Life sciences study design

All studies must disclose on these points even when the disclosure is negative.

Sample size	Sample size was determined based on previous publications on similar experiments to ensure confident statistical analyses.
Data exclusions	No data were excluded from analyses in the experiments.
Replication	All experiments were repeated at least two or three times and reliably produced.
Randomization	Plant materials used in the study were collected randomly.
Blinding	Investigators were not blinded to plant genotypes during experiments. The research materials are plants so the blinding design is not applicable to this system. Experiment results are not subjective.

Reporting for specific materials, systems and methods

We require information from authors about some types of materials, experimental systems and methods used in many studies. Here, indicate whether each material, system or method listed is relevant to your study. If you are not sure if a list item applies to your research, read the appropriate section before selecting a response.

Materials & experimental systems

n/a	Involved in the study	
☐	☒	Antibodies
☒	☐	Eukaryotic cell lines
☒	☐	Palaeontology
☒	☐	Animals and other organisms
☒	☐	Human research participants
☒	☐	Clinical data

Methods

n/a	Involved in the study	
☒	☐	ChIP-seq
☒	☐	Flow cytometry
☒	☐	MRI-based neuroimaging

Antibodies

Antibodies used

Commercial antibodies used:
α-HA-Peroxidase (Roche, Cat #12013819001, 1:3000)
α-FLAG-Peroxidase (Sigma-Aldrich, Cat #A8592, 1:3000)
α-GFP (Roche, Cat #11814460001, 1:3500)
α-His-Peroxidase (Roche, Cat #11965085001, 1:2000)
α-Mouse IgG, HRP-linked antibody (Cell Signaling, Cat #7076, 1:10000)
α-Rabbit IgG, HRP-linked antibody (Cell Signaling, Cat #7074, 1:10000)

Custom-made antibodies used:
α-BAK1 (GenScript, 1:1000)

Validation

The validation statements of commercial antibodies are available from the manufactures:
α-HA-Peroxidase (https://www.sigmaaldrich.com/US/en/product/roche/12013819001)
α-FLAG-Peroxidase (https://www.sigmaaldrich.com/US/en/product/sigma/a8592)

α-GFP (https://www.sigmaaldrich.com/US/en/product/roche/11814460001)
α-His-Peroxidase (https://www.sigmaaldrich.com/US/en/product/roche/11965085001)
α-Mouse IgG, HRP-linked antibody (https://www.cellsignal.com/products/secondary-antibodies/anti-mouse-igg-hrp-linked-antibody/7076)
α-Rabbit IgG, HRP-linked antibody (https://www.cellsignal.com/products/secondary-antibodies/anti-rabbit-igg-hrp-linked-antibody/7074)
The validation of the custom-made antibodies: α-BAK1 (reference 64)
