# Supplementary figures and images for: The Arabidopsis MIK2 receptor elicits immunity by sensing a conserved signature from phytocytokines and microbes
Source: Nat Commun. 2021 Sep 17;12:5494. doi: 10.1038/s41467-021-25580-w (PMC8448819; doi:10.1038/s41467-021-25580-w)

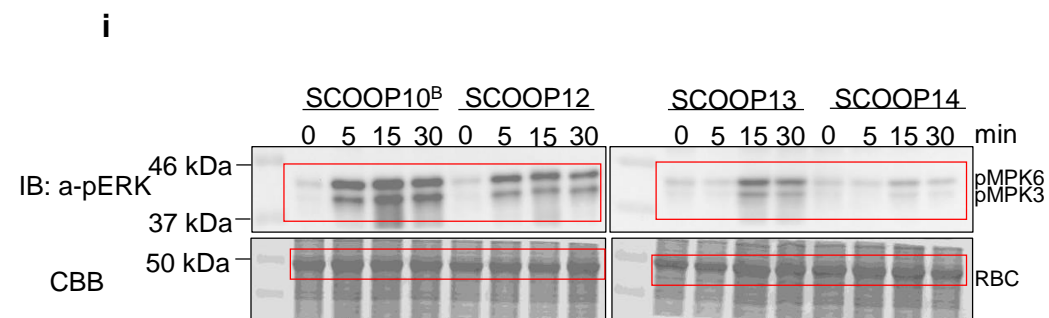

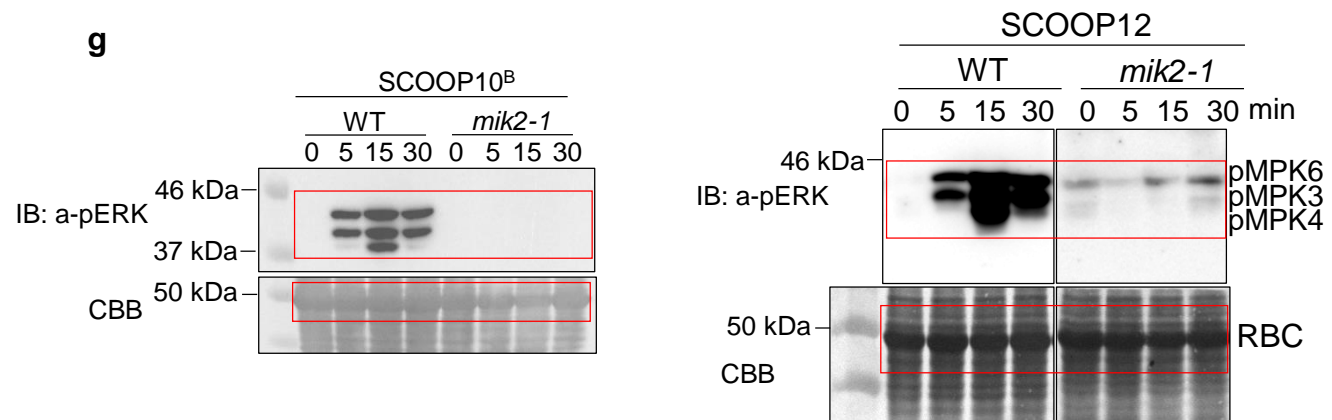

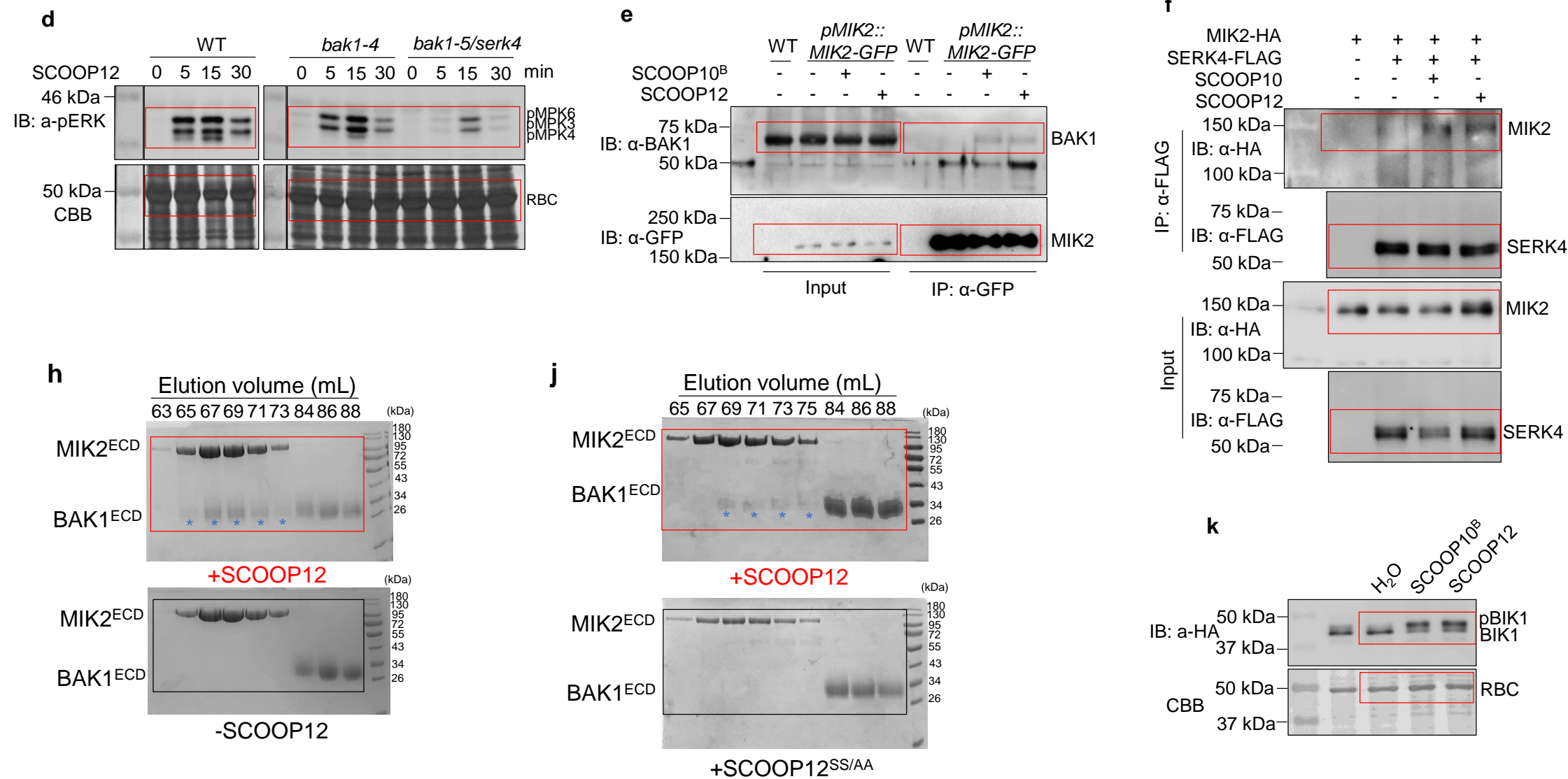

Source data Figure 5

**f**

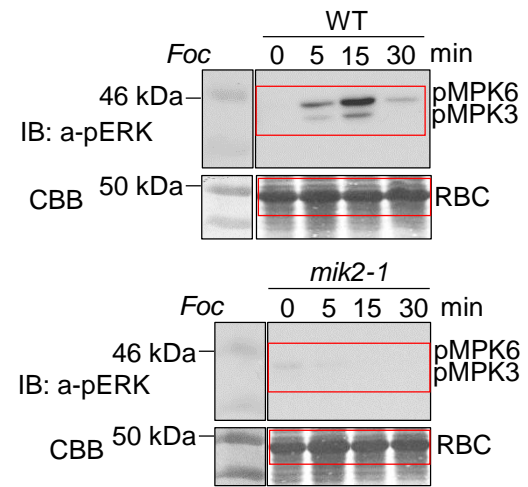

**j**

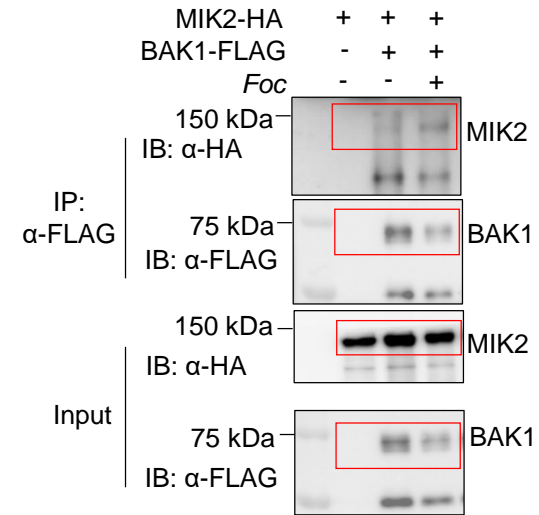

**d**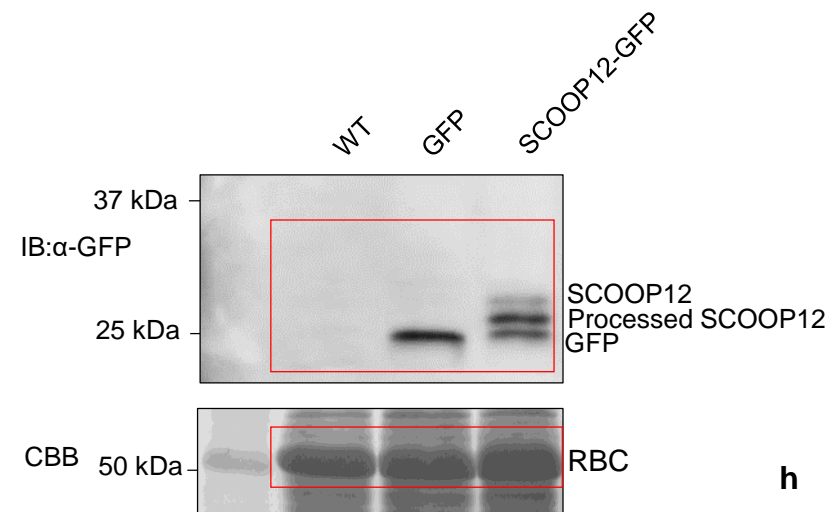**e**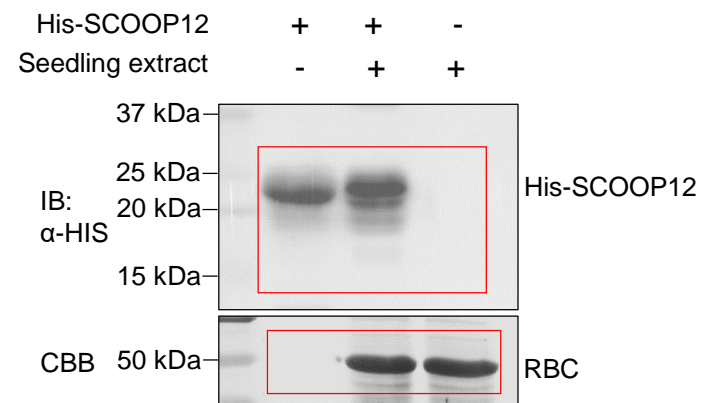**h**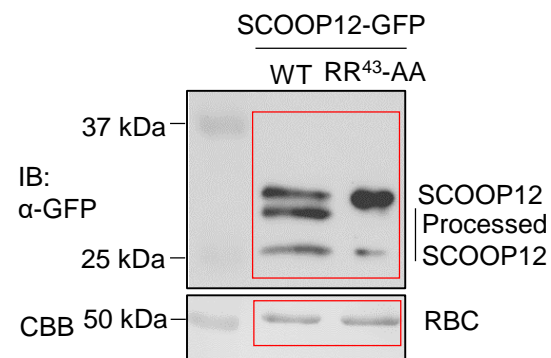**i**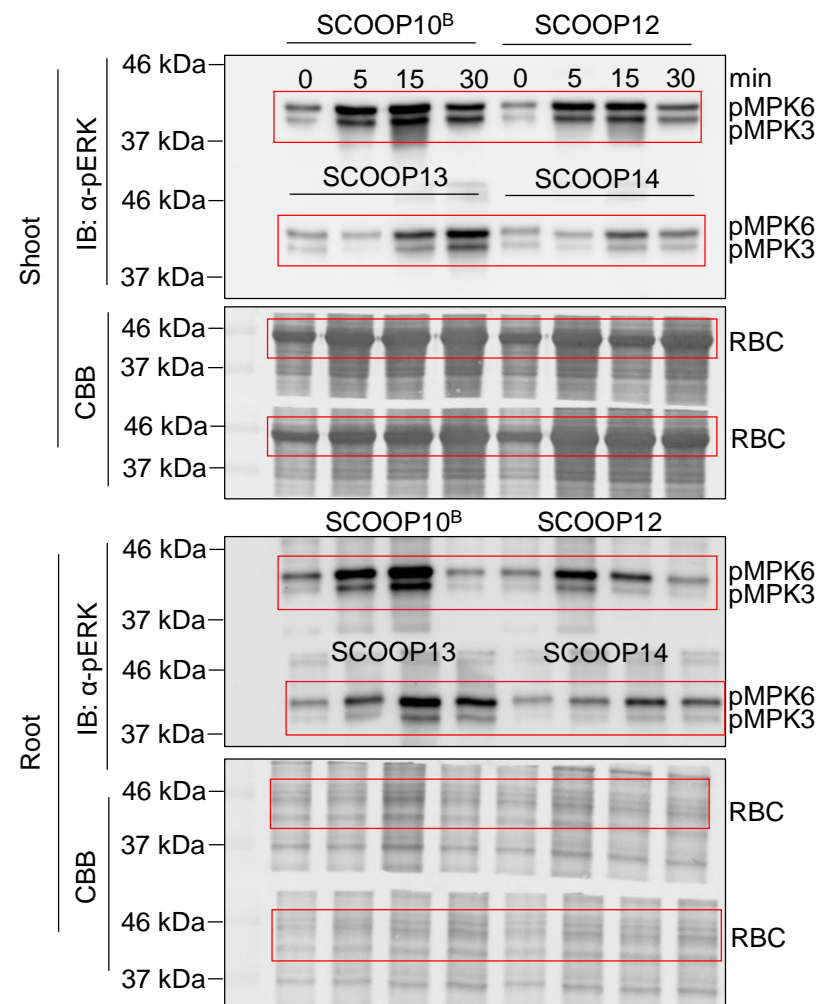

**c**

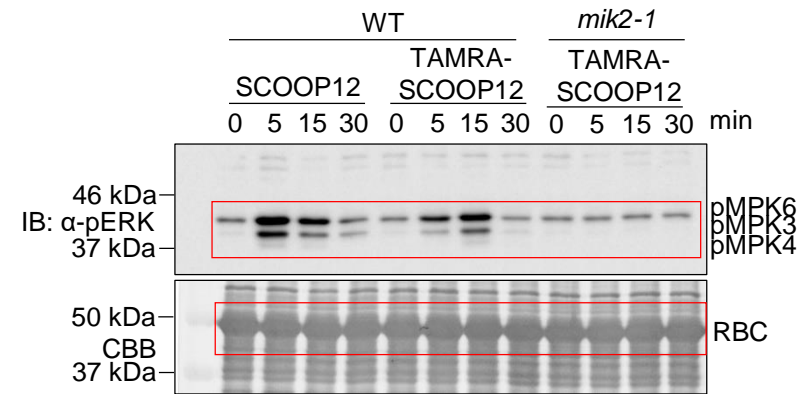

**a**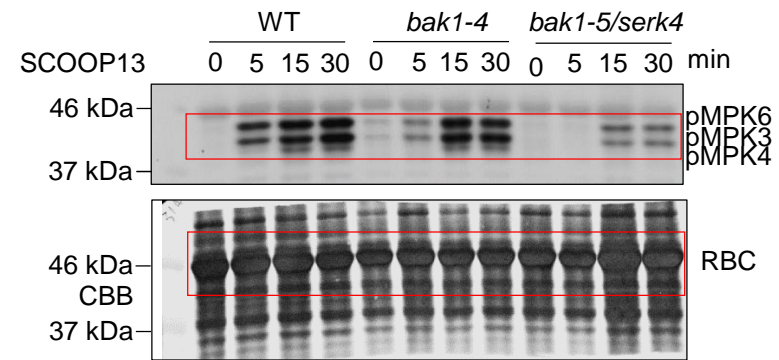**b**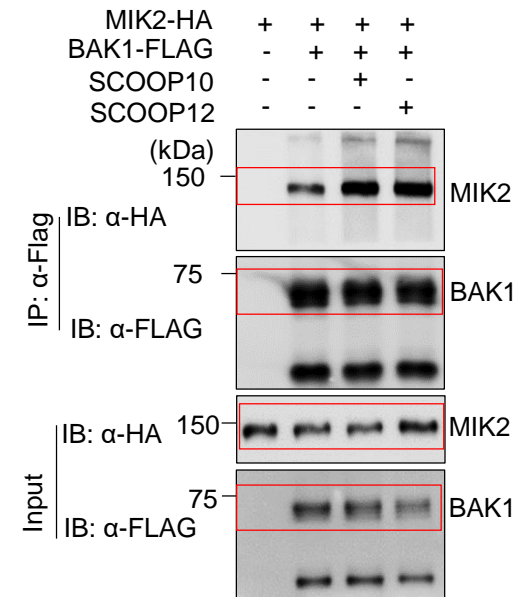

Supplement: Supplementary file 9 — Source Data [file 41467_2021_25580_MOESM9_ESM.zip › 282648_3_data_set_5818975_qxsrqh/Source data of gel blots.pdf]
